# Innate Lymphocytes in Psoriasis

**DOI:** 10.3389/fimmu.2020.00242

**Published:** 2020-02-21

**Authors:** Barbara Polese, Hualin Zhang, Bavanitha Thurairajah, Irah L. King

**Affiliations:** ^1^Meakins-Christie Laboratories, Department of Microbiology and Immunology, McGill University Health Centre Research Institute, Montreal, QC, Canada; ^2^Meakins-Christie Laboratories, Department of Medicine, McGill University Health Centre Research Institute, Montreal, QC, Canada

**Keywords:** innate, psoriasis, lymphocyte, skin, disease

## Abstract

Skin is a fundamental component of our host defense system that provides a dynamic physical and chemical barrier against pathogen invasion and environmental insults. Cutaneous barrier function is mediated by complex interactions between structural cells such as keratinocytes and diverse lineages of immune cells. In contrast to the protective role of these intercellular interactions, uncontrolled immune activation can lead to keratinocyte dysfunction and psoriasis, a chronic inflammatory disease affecting 2% of the global population. Despite some differences between human and murine skin, animal models of psoriasiform inflammation have greatly informed clinical approaches to disease. These studies have helped to identify the interleukin (IL)-23-IL-17 axis as a central cytokine network that drives disease. In addition, they have led to the recent description of long-lived, skin-resident innate lymphocyte and lymphoid cells that accumulate in psoriatic lesions. Although not completely defined, these populations have both overlapping and unique functions compared to antigen-restricted αβ T lymphocytes, the latter of which are well-known to contribute to disease pathogenesis. In this review, we describe the diversity of innate lymphocytes and lymphoid cells found in mammalian skin with a special focus on αβ T cells, Natural Killer T cells and Innate Lymphoid cells. In addition, we discuss the effector functions of these unique leukocyte subsets and how each may contribute to different stages of psoriasis. A more complete understanding of these cell types that bridge the innate and adaptive immune system will hopefully lead to more targeted therapies that mitigate or prevent disease progression.

## Introduction

The skin is the largest barrier organ. The most superficial layer of mammalian skin consists of an avascular, stratified epithelial layer that provides a physical and chemical barrier to environmental insults, is responsible for hair formation and supports a diverse commensal microbial community that promotes colonization resistance to invasive pathogens. Underlying the epidermis is the dermis composed of a fibroblast network providing structure for a complex neurovascular system that regulates heat transfer, pain sensation, and host defense (1). The epidermis and dermis harbor unique leukocyte subsets that are not only central to cutaneous immunity, but also contribute to basic skin physiology including wound healing, hair follicle cycling, and lipid production by sebaceous glands. Given the intimate relationship between immune-structural cell interactions, it stands to reason that aberrant communication within this compartment can lead to altered host defense mechanisms and/or dysregulated skin inflammation and disease. One of the most common cutaneous inflammatory diseases is psoriasis. Affecting between 2 and 5% of the adult population in developed countries, psoriasiform inflammation varies in severity but is most commonly characterized by red, scaly plaques across the surface of the body in a form referred to as psoriasis vulgaris. Although the etiology of psoriasis has not been identified, both environmental and genetic factors have been shown to contribute to incidence and severity of disease (1–3). Importantly, psoriasis is associated with comorbidities such as atherosclerosis and metabolic syndrome suggesting systemic dysregulation of the immune response in these patients providing further motivation for understanding disease pathogenesis (1, 4). Despite some differences between human and rodent skin, animal models of “psoriasiform” inflammation have been instrumental in identifying the immunological mechanisms underlying psoriasis development. For example, the models described in more detail below have helped to determine the interleukin (IL)-23/IL-17 axis as central to disease progression (1, 5). The essential role of these cytokines has been validated by the clinical efficacy of humanized monoclonal antibodies targeting TNFα, IL-23, IL-17A, and the IL-17 receptor (6, 7). However, these treatment approaches have limitations. First, they are not curative; symptoms reappear upon cessation of treatment. Thus, biologics must be given throughout the patient's lifetime. Second, the IL-23/IL-17 immune axis plays an important role in protection against cutaneous pathogens such as *Candida* and pathobiotic *spp*. of *Staphylococcus areus* (8, 9), thus raising questions about the long-term use of these treatments regarding susceptibility to infection. Furthermore, these biologics do not specifically target the skin and may compromise host defense at other barrier sites such as the intestine. Therefore, further investigation into the initiating factors that drive psoriatic disease will not only enrich our knowledge of skin biology in general, but lead to more targeted, tissue-specific treatments for this chronic inflammatory disease.

The recent discovery of immune cell subsets that are resident to the skin such as γδ T cells and innate lymphoid cells (ILCs) has prompted a growing interest in how these and other better known cell types that blur the separation between the innate and adaptive immune system such as Natural Killer (NK) cells and NKT cells contribute to psoriasiform inflammation. Indeed, these cells serve as acute sensors of infection and tissue injury without the need for specific recognition of antigen. While these properties have likely evolved to respond rapidly to tissue changes, their non-specific activation requirements leave them susceptible to hyperreactive responses against innocuous stimuli. In this review, we describe the diversity of innate lymphocyte lineages present in the skin and our current understanding of how each subset contributes to the pathogenesis of psoriatic disease.

## The Cutaneous γδ T Cell Compartment

Of the innate T lymphocytes in the skin, γδ T cells, defined by expression of gamma (γ) and delta (δ) TCR subunits, are the most studied. Their innate classification comes from two main characteristics: first, the repertoire of γ and δ chains possess less diversity than their more classical αβ TCR counterparts. Second, γδ T cells do not require TCR engagement in order to expand and exert their effector functions. Rather, cytokines alone are sufficient to endow γδ T cells with cytotoxic and cytokine-producing ability (10).

**Figure 1:**
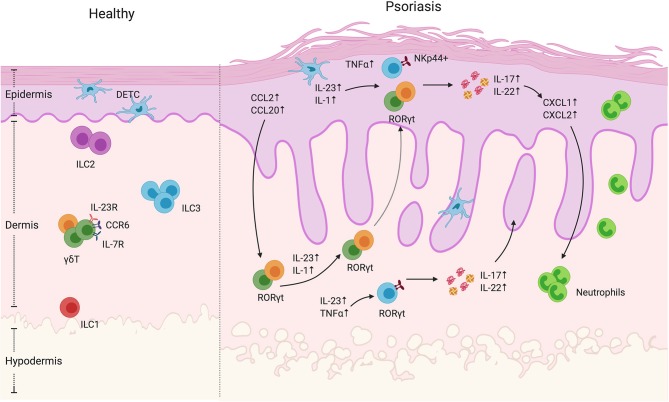
γδ T cells and ILCs in psoriatic skin. Diverse subsets of γδ T cells colonize the skin. Under homeostatic conditions, the mouse epidermis contains dendritic epidermal T cells, which are a monoclonal population of Vγ5^+^ cells. The dermis contains Vγ4^+^ and Vγ6^+^ γδ T cells enriched for expression of IL-23R, CCR6, and IL-7R. In mouse models of psoriasiform inflammation, activated keratinocytes produce chemokines such as CCL2 and CCL20, which subsequently recruit dermal γδ T cells to the epidermis. Among these γδ T cells, there is a subgroup that express the transcriptional factor RORγt, that are capable of producing IL-17 and IL22 upon IL-1 and IL-23 stimulation. Both mouse and human studies have shown that, upon cytokine stimulation, dermal-derived γδ T cells secrete IL-17 and IL-22 that drives keratinocyte hyperplasia, neutrophil recruitment and disease progression. ILCs are also present in the healthy skin. They are divided into three groups based on transcription factor expression and effector functions. Under steady-state conditions, ILC2 are the largest population. ILC3 are currently thought to be the dominant population that contribute to disease progression. In human skin lesions, NKp44^+^ ILC3s are able to produce IL-22 and IL-17 that exacerbate disease progression.

In mice, γδ T are usually distinguished based on the γ chain expression. It is worth mentioning that two nomenclatures are often used but rarely specified in the literature, namely the Heilig and Tonegawa vs. the Garman classification. In this review, we will use the Heilig and Tonegawa nomenclature only, which includes the Vγ1–Vγ7 subtypes (11). Each subtype has a propensity to localize to specific organs as well as exert unique effector functions. Their development and migration to the epithelial tissues starts during fetal life (12–14) with consecutive waves associated with different γδ T subsets migrating from the thymus to their specific tissue (10, 15). From day E13, the Vγ5 subtype is produced in the thymus and migrates to the epidermis (Figure 1). Vγ5 γδ T cell development is exclusively fetal and occurs only in mice. These cells are called dendritic epithelial T cells (DETC) due to their morphology, are non-migratory and are maintained by self-renewal (16, 17). As DETC seem to be most relevant for maintaining skin homeostasis and wound repair and have been reviewed extensively elsewhere, we will not be discussing this subset further. On the other hand, Vγ4 and Vγ6 subtypes constitute the dermal γδ T cell compartment (Figure 1). Unlike DETCS, dermal γδ T cells are motile with Vγ6^+^ cells seeding the dermis during fetal life and Vγ4^+^ cell recruitment limited to the first days of life (18). Accordingly, the dermal γδ T cell compartment can be replenished after irradiation, but only if neonatal thymocytes are transferred (19).

Vγ usage is also associated with a specific effector function profile. In fact, γδ T cells can be largely defined based on their expression of lineage-restricted transcription factors and effector functions. The most prominent subsets include IFNγ (γδ1) and IL-17 producing γδ T cells (γδ17) that rely on the transcription factors T-bet and RORγt, respectively, for their differentiation (20). Interestingly, however, γδ T cell effector functions are uniquely imprinted in the thymus where SOX13 drives γδ T cell lineage commitment and subsequent TCR dependent and independent mechanisms that dictate effector functions (21). For example, CD27 is a thymic determinant of γδ T cell fate by promoting γδ1 over γδ17 cells and inducing IFNγ-associated genes (22). Additionally, strong TCR engagement favors IFNγ-producing γδ T development (23) while limiting γδ17 development (24). As a result, IFNγ and IL-17-producing γδ T subsets can be identified on the basis of CD27 and CCR6 expression, amongst other markers (22, 25). Dermal Vγ4^+^ and Vγ6 ^+^ γδ T cells express several hallmarks similar to Th17 cells including RORγt, IL-7R, CCR6, and IL-23R expression as well as ability to produce IL-17 (19, 26). They can be stimulated by IL-23, which leads to their expansion and IL-17 production (26) (Figure 1). Dermal γδ T cells have been associated with immunosurveillance functions. In the context of mycobacterial infections, they have been shown to be the dominant source of IL-17 and their absence was correlated with diminished immune response to BCG immunization (27). Furthermore, IL-17 production by dermal γδ T can be stimulated by various microbe-derived products (26), further emphasizing their immune sentinel role. As Vγ6^+^ cells are rarely found in secondary lymphoid organs, MacKenzie et al. suggested that this subset might have specifically evolved for immunosurveillance of non-lymphoid tissues while the more migratory, lymphoid organ-skewed Vγ4 subset might serve as a pool that is rapidly mobilized to barrier sites following challenge (28).

In humans, γδ T cells are usually distinguished based on δ chain expression including Vδ1, Vδ2, and Vδ3 (i.e., Vδ1-Vδ2-) subtypes. Vδ1 cells seed barrier tissues while Vδ2 and Vδ3 are observed in the blood of healthy patients (29). Similar to murine γδ T cells, human γδ T cells are potent cytokine-producing cells, but the regulatory mechanisms are less understood. Unlike murine γδ T cells, human γδ T cells are more dependent on TCR engagement for activation and appear to produce a greater diversity of effector cytokines. For example, human γδ17 cell differentiation, which likely occurs in the periphery since they are absent from the human mature thymus (30), requires IL-23 and TCR activation. Furthermore, Vγ9Vδ2 cells that represent the majority of the Vδ2 subset, exhibit remarkable heterogeneity in term of surface markers and cytokine production. These plastic cells are able to produce IFNγ, IL-4, or IL-17, which contrasts with murine γδ T cell commitment (31).

As opposed to mice, human γδ T cells are rare in the skin with Vδ1-expressing cells being the dominant subtype observed in healthy skin, mainly in the dermis. With the help of αβ T cells (16), Vδ1 seem to recapitulate the role of DETC given that they present a restricted repertoire (32), can be observed in the epidermis, produce keratinocyte growth factors and exert anti-tumor activity (31, 33). Vδ1^+^ cells are also usually associated with IFNγ production and a cytotoxic profile (34). Notably, human dermis-derived γδ T cells have been shown to produce IL-17A. In fact, Cai et al. found IL-17-producing γδ T cells to be enriched in psoriatic skin lesions. However, the full repertoire of cutaneous γδ T cells has yet to be investigated (26).

### γδ T Cells Are Major Contributors to Murine Psoriasiform Inflammation and Implicated in Human Disease

Two mouse models of cutaneous inflammation are most commonly used to study the mechanisms underlying psoriasiform inflammation. The imiquimod (IMQ) model that consists of topically applying a TLR7/8 agonist emulsified in a cream or intradermal injection of recombinant IL-23 (5, 35). Both approaches lead to epidermal hyperplasia, parakeratosis, and expansion of rete ridges, all features of psoriasiform inflammation (36). These preclinical models have been shown to depend on the presence of IL-17 for fulminant inflammation and motivated clinical trials the development of neutralizing antibodies targeting IL-23, IL-17A, or IL-17RA (the receptor for both IL-17A and IL-17F) (5–7, 26). The incredible clinical success of these biologics has validated these models and led to further investigation into the cell types driving IMQ and IL-23-induced inflammation (6, 7). Importantly, both models revealed decreased inflammation and psoriasiform symptoms inflammation in mice genetically lacking γδ T cells (TCRδ^−/−^) compared to mice deficient in αβ T cells (TCRα^−/−^) mice (26, 37). Importantly, TCRδ^−/−^ mice reconstituted with Vγ4 and Vγ6 subpopulations restored disease susceptibility (18). Similarly, selective depletion of Vγ6^+^ or Vγ4^+^ γδ T cells using antibody-mediated or genetic depletion approaches indicate that both subsets are necessary and sufficient for IMQ-induced inflammation (38, 39). Interestingly, Vγ4^+^ γδ T cells have been shown to have memory-like capacity. Indeed, two papers have demonstrated that this γδ T cell subset persists in the skin after termination of IMQ treatment and exhibits classical features of memory cells upon secondary IMQ challenge (i.e., a more rapid response with greater magnitude) in the same area or even distant sites (40, 41). Ramirez-Valle et al. further demonstrated that the migration and recruitment to distant sites was mediated via CCR2 signaling (41). They showed that IMQ-activated Vγ4^+^ T cells expressed less CCR6 than unchallenged γδ T cells and that the former subset demonstrated increased responsiveness to IL-1. Downregulation of CCR6 was unexpected as it was previously shown that both models of psoriasis induce CCL20 (42), the chemokine recognized by CCR6, and that a CCL20/CCR6 axis was essential for disease progression (38, 43) (Figure 1). Induction of CCL20 leads to dermal IL-17^+^γδ T cell recruitment into the epidermis, exacerbating inflammation. Accordingly, an anti-CCL20 antibody treatment reduced IL-23-induced inflammation by decreasing the γδ T trafficking into the epidermis (42). In the latter study, the source of chemokine secretion was not identified but it has been demonstrated that IL-1β can stimulate keratinocyte production of CCL2 and CCL20, which might impact γδ T cell recruitment (18). In addition, activated dermal γδ T cells increase expression of X-linked IL-1 receptor accessory protein-like 1 (IL1RAPL1) which promotes a feedforward system inducing more IL-17 production by these cells. IL-38, a cytokine of the IL-1 family secreted by keratinocytes at steady state, is able to restrict γδ T cell activity by inhibiting IL1RAPL1 on the surface of γδ T cells (44). Accordingly, the levels of IL-38 secreted by the keratinocytes is decreased in psoriatic lesions as well as in mouse skin following IMQ treatment (44, 45). These results underline the loop that exacerbates psoriasis, where inflammation induces keratinocytes secretion of chemokines, which in turn triggers γδ T cell recruitment. The pro-inflammatory environment leads to cytokine production by γδ T cells, which promotes keratinocyte hyperproliferation and epidermal thickening.

Although γδ T cells are capable of cytotoxic activity, their potent cytokine production seems to play a dominant role in psoriasiform inflammation (Table 1). In the IMQ model, both IL-17 and IL-22 production by RORγt ^+^ γδ T cells, Vγ4^+^ cells in particular, is greatly increased (37) (Figure 1). Consistent with these results, IL-17R deficient mice showed reduced and delayed signs of psoriasiform inflammation such as ear thickness and erythema after IMQ treatment (56). However, disease was not completely abolished in IL-17R deficient mice and increased levels of TNFα, IL-6, and IL-22 as well as IL-17-producing cells were observed in the skin. This demonstrates the importance of IL-17 signaling for psoriasiform inflammation, but also suggests an alternative pathway for IMQ-induced inflammation. Similarly, IMQ-induced inflammation was strongly reduced in mice with a keratinocyte-specific deletion of the IL-17 receptor (57). In another study using the IMQ model, the main producer of IL-22 was also γδ T cells. However, in Rag-deficient mice that lack mature T and B cells, levels of IL-22 in response to IMQ remained elevated suggesting an alternative source of cutaneous IL-22 (50). Although it was shown that, in addition to IL-17, IL-22 is required for IL-23 induced inflammation, the failure of clinical trials using anti-IL-22 antibodies have kept the focus on the effector functions of IL-17 and its associated family members. In fact, a recent report showed that IL-17E (better known as IL-25) signaling via IL-17RB also plays an important role in IMQ-induced psoriasiform inflammation (51). This work was recently followed up by studies demonstrating that IL-17A can signal via an alternative receptor, IL-17RD, to drive psoriasiform inflammation (58). To conclude, γδ T cells are major contributors to murine psoriasiform inflammation via the production of IL-17 and IL-22 (Figure 1, Table 1). The Vγ4 subtype is particularly implicated in the disease due to its quick cytokine response, migration capacities and long-lasting memory capacity.

**Table 1:** Cytokines produced by innate immune cells during psoriasis.

**Cytokine**	**Cell types**	**References**
IL-17A	γδT cell, ILC3, NK cell	(18, 26, 37, 38, 46–49)
IL-22	γδT cell, ILC3, NK cell	(37, 47, 50)
IL-25 (IL-17E)	γδT cell	(51)
IFNγ	NK and NKT cell	(52–55)
TNFα	NK and NKT cell	(53)

Such as in mice, γδ T cells are expanded in human psoriatic skin and produce IL-17A (26) (Table 1). A population of Vγ9Vδ2^+^ cells that express IL-17A, IFNγ, TNFα and CCR6 has been specifically observed in human psoriatic lesions (59). These cells were able to activate keratinocytes and stimulate chemokine, cytokine and defensin production. Laggner et al. also showed that Vγ9Vδ2^+^ cells were increased in psoriatic skin compared to healthy skin and, even more, increased in lesional skin compared to non-lesional skin of the same patients (59). In addition, Vγ9Vδ2^+^ cells were reduced in psoriatic patient blood. Finally, they showed a negative correlation between blood levels of Vγ9Vδ2^+^ cells and psoriasis severity. These results suggest that the Vγ9Vδ2^+^ population is recruited from the peripheral blood to the skin where they activate keratinocytes and contribute to psoriasis development. On the other hand, it has been recently shown that the majority of IL-17A producing T cells observed in human psoriatic lesions are oligoclonal αβ T cells and not γδ T cells (60). Furthermore, mast cells have been shown to produce IL-17A and IL-22 in human psoriatic plaques (61). The diverse subsets previously found to be expressing and/or producing IL-17 cytokines in human psoriasis and disparate results between groups continues to fuel a controversy over the most relevant cytokine-producing cells for psoriatic disease development and progression. Longitudinal studies using large, diverse patient cohorts may help reconcile these differences.

## The Innate Lymphoid Cell Skin Population

ILCs are bone marrow-derived tissue-resident lymphocytes that, although arising from common lymphoid progenitors, do not express rearranged antigen-specific receptors. ILC nomenclature is largely analogous to CD4^+^ T helper effector cell subsets: ILC1s express the transcription factor T-bet and secrete IFNγ, ILC2s express GATA3 and produce the Th2 cytokines IL-5 and IL-13 and ILC3s express RORγt and secrete IL-17 and IL-22. Although ILCs are thought to be largely tissue-resident cells (62), ILCs have been detected in the circulation that express high levels of cutaneous leukocyte-associated antigen (CLA), a skin homing marker (63). In both mice and humans, all three groups of ILCs have been observed in the skin with ILC2s being the largest population (63–65). Furthermore, a study examining the cutaneous ILC population in mice showed that different layers of the skin are populated differentially by ILCs: the epidermis is mainly populated by ILC3s, the subcutaneous layer is populated by ILC2s and the dermis contains both ILC2s and ILC3s (66) (Figure 1). However, the signals that result in the differential homing of ILCs in the skin and whether this is representative of human ILC populations is not completely understood. ILC1s, although present in the skin, are a rare population with unknown functions. Although sharing several features with natural killer (NK) cells, ILC1s do not exert cytotoxic activity—lack perforin and granzyme expression—and do not express traditional NK cell antigens such as CD56, CD16, or CD94. However, the cytokine profile of ILC1s, most notably IFNγ, resembles NK cells and has been shown to play a role in the protection against intracellular pathogens (62, 67, 68). As ILC1s are thought to contribute to Crohn's disease and inflammation in a mouse model of colitis (69, 70), they could potentially play similar roles in the skin both in terms of protection as well as autoimmune-like pathology, however this has not been thoroughly investigated. ILC2s on the other hand are much more common in the skin and are thought to play a role in maintaining skin homeostasis. For example, ILC2s have been shown to promote wound healing in the skin through the production of IL-13 (71, 72). Skin-resident ILC2s can also produce high levels of amphiregulin, a molecule regulating wound healing (73). In dermatitis, amphiregulin has been shown to play a role in wound healing by acting as an epidermal growth factor receptor (EGFR) ligand (74). However, other evidence indicates the involvement of ILC2s in allergic-type or type 2 inflammation of the skin, namely atopic dermatitis likely through dysregulated production of type 2 cytokines such as IL-5 and IL-13 (73, 75, 76). Lastly, ILC3s are one of the subtypes of immune cells in the skin capable of producing IL-17A and IL-22 and are therefore of specific interest when discussing psoriasis (Figure 1).

### ILC3s Are Observed in Human Psoriatic Skin and Correlate With Disease Severity

While ILC3s seem to play a role in the development and maintenance of psoriasis, the role of ILC1 and 2 subsets is a matter of debate (Figure 1). Some groups found a reduction in ILC2 numbers in psoriatic patients (64) while others saw no difference in frequencies. Notably, different methods of tissue processing from skin biopsies in these studies may explain the differences (63–65). Given that ILC2s are known to play a role in maintaining skin homeostasis and wound healing (71, 72), they may also be playing a protective role during the development of psoriasis. Second, these studies did not indicate involvement of ILC1s (63–65). However, one group reported a significant increase in the number of ILC1s in psoriatic skin (77); this latter group detected the number of ILCs using imaging of whole skin whereas the other groups performed flow cytometry which may explain the difference. Since ILC1s in the gut seem to play a role in inflammatory pathologies, it is possible that ILC1s may also be paying a role in inflammatory pathologies in the skin such as psoriasis. When looking at the cells in circulation, both healthy individuals and psoriatic patients have a similar mean frequency of ILCs in total peripheral blood mononuclear cells (PBMCs) (65). However, there seems to be an overall increase of ILCs in psoriatic skin (Figure 1). This increase in ILCs is mainly due to an increase of ILC3s (63, 64, 77). NKp44 has been associated with pro-inflammatory functions in ILC3s, its activation leading to TNFα production (78). ILC3s in the skin of healthy patients were shown to be mainly NKp44–, whereas NKp44^+^ ILC3s were barely detectable in the skin and blood (63). NKp44 expression is induced in NKp44-ILC3s upon IL-1β and IL-23 stimulation, cytokines commonly present in psoriatic inflammation (63) (Figure 1). In psoriasis patients, the levels of NKp44^+^ ILC3 but not NKp44– ILC3s were increased in the blood, lesional, and non-lesional skin. Furthermore, psoriasis severity as measured by the PASI scoring system positively correlated with the number of cutaneous NKp44^+^ ILC3s (63–65). These data suggest that the amount of NKp44^+^ ILC3s in the blood or the skin can potentially be used as a biomarker for disease severity. Furthermore, ILCs in psoriatic skin were seen to be in close proximity to T cells, suggesting a crosstalk between ILCs and T cells during the development of psoriasis (77). Given the innate features of ILC3s and their largely tissue-resident nature, these cells may contribute to the initiation of psoriatic inflammation. Indeed, ILC3s alone were able to induce psoriasis in a human skin xenotransplant mouse model to a degree similar to αβ T cells (79). Furthermore, patients with psoriatic arthritis, a disorder with similar features of psoriasis but with joint involvement, also had an increased ILC3: ILC2 ratio (80).

As mentioned above, IL-17 producing γδ T cells have been shown to be important drivers of IMQ-induced inflammation (81). However, it has been shown that Rag-deficient mice are still susceptible to psoriasiform inflammation via IMQ (37, 43), indicating that cells other than T cells play a role in the pathogenesis. Using Rag1/IL-2R deficient mice lacking T cells and ILC, Pantelyushin et al. showed that RORγT^+^ γδ T cells and RORγt^+^ ILC contribute to IMQ-induced psoriasiform inflammation (37). Furthermore, anti-TNFα or TNFα inhibitor treatment has been demonstrated to be a very effective treatment for psoriasis (82). TNFα plays a role in psoriasis development by synergizing with IL-23 to induce IL-17 producing cells, including ILC3s (46). Individuals undergoing successful anti-TNFα (adalimumab) treatment for psoriasis had a reduction in the number of pathogenic NKp44^+^ ILC3s and an increase in NKp44– ILC3s in the circulation (65), suggesting that a major role of TNFα in the pathogenesis of psoriasis includes potentiating pathogenic ILC3s. However, it was elegantly demonstrated that γδ17 were non-redundant effector cells in murine skin pathology (81). Indeed, when γδ17 cells were deleted from birth, they were replaced by IL-17 producing ILC3s that promoted IMQ-induced inflammation. However, acute depletion of γδ17 cells did not lead to ILC3 accumulation and mice remained resistant to psoriasiform inflammation. In summary, ILC2s appear dominant in healthy skin whereas NKp44^+^ ILC3s are the major ILC subset associated with psoriatic disease. Although ILC3s and γδ17 cells may play overlapping roles in murine models of psoriasis, more studies are needed to discern their relative contributions to human disease.

## Cutaneous NK and NKT Cells

**Figure 2:**
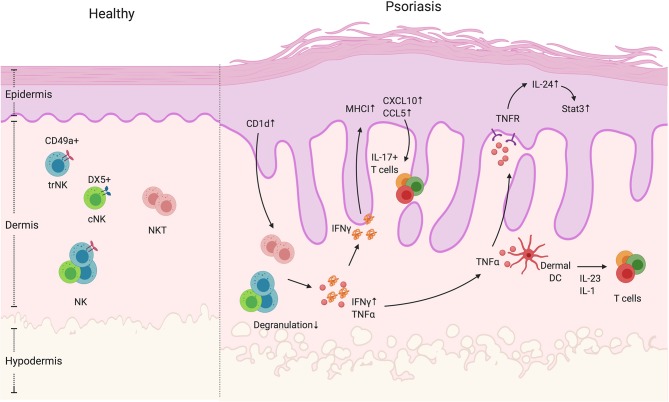
NK and NKT cells in psoriatic skin. NK and NKT cells are innate immune cells that have cytokine-producing and cytotoxic functions. They both reside in the dermis. The NK cells can be divided into two groups, namely cNK and trNK cells, based on the receptors CD49a and DX5. Unlike NK cells, NKT cells also express an antigen-specific TCR that recognizes glycolipid through CD1 presentation by antigen-presenting cells. In psoriatic skin lesions, NK and NKT cells are rare. However, CD1d expression is reported to be elevated in keratinocytes in inflamed skin. In addition, NK and NKT cells have decreased degranulation ability, but display increased IFNγ production. High IFNγ production can contribute to an increase in keratinocyte-derived chemokines such as CXCL10 and CCL5, and the elevated expression of MHCI, both of which increase cell recruitment and presentation of autoantigens. In addition, NK and NKT cells produce TNFα that activate keratinocytes in an IL-24/Stat3-dependent manner as well as indirectly enhance dermal IL-17^+^T cell activation by facilitating dendritic cells to produce IL-1 and IL-23.

Natural Killer (NK) cells are a group of innate immune cells with both cytotoxic and cytokine producing effector functions and have been recently classified as one of two ILC1 subsets (83, 84). Through germ-line encoded activating and inhibitory receptors, NK cells can respond quickly following activation, releasing pro-inflammatory cytokines particularly IFNγ, chemokines, or specialized cytotoxic granules to infected or tumor cells (85). In human and mice, there are two distinct populations of NK cells, circulating NK cells (cNK, CD49a^+^CD103^−^ or CD56^dim^CD16^+^ in human and CD49a^−^DX5^+^ in mice) and tissue-resident NK cells (trNK, CD49a^−^CD103^+^, or CD56^bright^CD16^−^ in human and CD49a^+^DX5^−^ in mice) (Figure 2); both can induce cytotoxicity and produce cytokines (86–89). Murine skin is composed of both trNK cells and cNK cells (87) (Figure 2). However, the cNK and trNK cells do not share the same development pathways. cNK cells are derived from the bone marrow, continue their maturation in the thymus and then the spleen, tonsils and lymph nodes (90, 91). In mice, the transcription factors T-bet and Eomes are required for the maturation of cNK cells (92). In humans, both T-bet and Eomes are co-expressed in mature cNK cells (93). T-bet is expressed at lower levels in cytokine-producing CD56^bright^(CD56^hi^CD16^−^) NK cells than the highly cytotoxic CD56^dim^ (CD56^lo^CD16^+^) NK cells, while CD56^bright^ NK cells have higher frequency of Eomes^+^ cells than CD56^dim^ NK cells (93), indicating that there is a gradual loss of Eomes expression during the development of CD56^bright^ cells to T-bet^hi^Eomes^+^ CD56^dim^ cells. trNK cells were first discovered in the murine liver, strictly require T-bet, Hobit and PLZF for their development, however do not express Eomes (87, 89). Murine liver trNK cells are capable of degranulation and produce similar IFNγ levels to cNK cells. However, both the liver IFNγ^+^ and degranulating trNK cells produce TNFα, which is rarely seen among responding cNK cells (87). Unlike mouse trNK cells, human liver trNK cells have high Eomes expression rather than T-bet (94). Of note, the features and developmental pathways of trNK cells differ from one organ to another. In the murine gut and dermis, the development of NKp46^+^CD3^−^ trNK cells is reported to be dependent on the transcriptional factor RORγt and RORγt^+^ trNK cells are capable of producing IL-22 (95). The origin of skin trNK cells is unclear, but murine studies show that skin trNK cells share some features with liver trNK cells, in terms of phenotype, function and developmental requirements. They are CD49a^+^DX5^−^ with no Eomes expression, and their development is dependent on IL-15 and IL-15R. Human CD56^bright^CD16^−^NK cells are present in the dermis at steady state and disease conditions such as psoriasis, while CD56^+^CD16^+^ cNK cells are rare (52, 96, 97). These CD56^bright^CD16^−^ dermal NK cells lack perforin and NKG2D expression but are capable of lysing melanoma cells after activation *in vitro* (97). Recently, studies have found IL-17 and IL-22 producing NK cells in both humans and mice, which indicates the potential for NK cell participation in the development of psoriasis (47–49).

Natural Killer T (NKT) cells are present in both human and mouse skin (Figure 2). However, the composition of NKT cells is not well-defined. In human allergic contact dermatitis, for example, NKT cells range from 1.72 to 33% of the T lymphocyte infiltrate and in human atopic dermatitis patients, the proportion of NKT cells in CD3^+^ T cells is ~5% (98, 99). In murine skin, they compose ~0.03% of total healthy skin cells and ~0.6% of total hyperplastic skin cells (100). NKT cells are a unique hybrid between αβ T cells and NK cells as they co-express an αβ TCR and NK cell lineage markers. NKT cells are divided into four categories with type 1 (referred to as invariant NKT cells) being the vast majority (101). Compared to conventional T cells, they express a semi-invariant TCR α chain (Vα14-Jα18 in mice and Vα24-Jα18 in human), which allows specific recognition of glycolipids presented on an atypical MHC Class I molecule, CD1 (102–104). α-galactosylceramide (α-GalCer), a compound derived from marine sponges, has a strong CD1d binding affinity and is a potent stimulant for iNKT cells. Potential endogenous ligands of NKT cells were previously believed to be glycosphingolipids (GSLs) and phospholipids that are derived from bacterial, plant, protozoan, and mammalian species. However, more recent studies suggest that NKT cell ligands are more diverse and not limited to GSLs (105, 106). Thus, the endogenous ligands of NKT cells are still being clarified. When stimulated with α-GalCer or its analogs, NKT cells rapidly produce pro- and anti-inflammatory cytokines including IFNγ, TNFα, IL-10, IL-4, IL-13, IL-17 and GM-CSF, and participate in the regulation of infection, autoimmunity, and tumor immunity (107). Unlike NK cells, NKT cells undergo positive and negative selection within the thymus, but emerge later in development than most other T cell subsets (108, 109). During the selection process, NKT cells are only selected when CD1 is expressed on double positive (CD4^+^CD8^+^) thymocytes, which segregates the NKT cell (CD161 low in human and NK1.1- in murine at this stage) from the conventional T cell developmental pathway (110–114). The transcriptional factors Ras, Mek, Fyn, and Ets1 are reported to participate in the development of murine NKT cells, and the cytokine IL-15 and its receptor IL-15R are important during NKT cell development (115–118). After selection, the immature human CD161low or murine NK1.1- NKT cells either stay in the thymus or migrate to peripheral tissues, where they undergo a maturation process with the upregulation of CD161 (human) or NK1.1 (murine) expression (108, 109). The transcription factor T-bet was shown to participate in the terminal maturation of NKT cells (119). Both mouse and human NKT cells can exert cytotoxicity and produce seemingly antagonistic IL-4 and IFNγ cytokines upon TCR stimulation (120, 121). However, cytokine production may be developmentally regulated as mature NKT cells produce high levels of IFNγ while IL-4 is dominantly produced by immature NKT cells (108, 109). Recent data showed that NKT cells can also secrete Th17-related cytokines such as IL-17A, IL-17F, and IL-22 (107, 122, 123). A murine CD4-NK1.1- NKT cell group, which is the precursor of CD4-NK1.1^+^ NKT cell, has been found to constitutively express RORγt and IL-23R and is a major source of IL-17^+^ NKT cells (107). In addition, α-GalCer-activated murine NKT cells, that can express RORγt and IL-17, but not IFNγ or IL-4, develop in a c-Maf dependent way. These IL-17^+^NKT cells are essential for inducing neutrophil-rich airway inflammation (122). In humans, even though RORγt^+^ T-betloPLZF- NKT cells are found in the circulating PBMCs, the IL-23R expression is almost completely absent on circulating NKT cells. These NKT cells show poor IL-17 release after IL-23 stimulation. However, TCR stimulation (e.g., α-GalCer or αCD3/CD28Ab) in the presence of IL-2, IL-23, IL-1β and TGFβ1, NKT cells successfully produce IL-17 but not IFNβ or TNFα (123). Interestingly, there are more IL-23R^+^ NKT cells in the PBMCs and joint compartment of Spondyloarthritis patients than healthy controls, showing an IL-17 signature (123), which suggests that NKT cells could participate in the development of psoriasis. Although cutaneous NKT cells are important for the anti-microbial response due to their ability to recognize the bacterial glycolipids via CD1d presentation (124), they may function differently in cutaneous diseases, a result that may depend on the microbial and/or self-antigen repertoire of the skin. It has been shown that large numbers of NKT cells can be recruited into human skin during contact dermatitis, producing mainly IFNγ (98, 99) however, results in animal studies are controversial. Murine NKT cells were previously reported to suppress this response by producing IL-4 and IL-13 in response to CD1d-presented haptens (125), while it was also reported that murine NKT cells enhance the contact sensitivity reaction (126–128). Different results may be explained by the animal model studied, which shape the NKT cell cytokine repertoire. Studies have found decreased number of circulating Vα24^+^ NK T cells in atopic dermatitis patients, and they produce both IL-4 and IFNγ (99, 129). NKT cells were also shown to suppress skin transplant rejection, through the production of IL-4 (130–132). To conclude, even though the proportion of NK and NKT cells is rare, they do participate in cutaneous immunity through diverse effector programs.

### NK and NKT Cells Are Rare in Psoriatic Skin

The role of NK and NKT cells in psoriasis development is not clear. Even though studies showing involvement of NK cells in psoriasis are rare, NK cells have been shown to be present in psoriatic skin. Human studies show that NK cells are recruited in psoriatic plaques, particularly in the dermis (52, 133) (Figure 2). The psoriatic lesion-isolated NK cells exhibited low degranulation ability. However, their cytokine-producing ability is dependent on the source of NK cells (52, 53). Ottaviani et al. observed higher IFNγ production by NK cells isolated from psoriatic lesions and showed that IFNγ was able to induce keratinocyte chemokine production (such as CXCL10 and CCL5) and MHC-I expression (52) (Figure 2, Table 1). Consistent with the human data, mice treated with IMQ had increased NK1.1^+^ cells in the skin, which suggests that either NK or NKT cells were recruited into the skin during psoriasiform inflammation (134). Another study showed that NK cells from PBMCs of patients with psoriasis vulgaris have reduced cytotoxicity and lower levels of pro-inflammatory cytokines IFNγ and TNFα (53). However, questions remain about NK cells in the context of psoriasis. Psoriasis was initially thought to be a IFNγ related disease but more recent studies—and the success of biologics targeting the IL-17 pathway—indicate a more dominant role for TNFα and IL-17 driven disease (1, 135, 136). As suggested above, TNFα and its associated receptors have been reported to be elevated in psoriatic lesions compared to non-lesional skin and TNF-R is abundantly expressed by keratinocytes (137, 138). It has been reported that TNFα signaling is involved in IL-24-induced psoriasis like inflammation in mice (139). In addition, both TNFα inhibitors and blocking antibodies show efficacy in alleviating psoriatic arthritis symptoms (140). Since both IFNγ^+^ and degranulating skin trNK cells produce TNFα (87), it is possible that skin NK cells participate in the progression of psoriasis by the production of TNFα rather than IFNγ. To address this question, TNFα production by NK cells in the skin of healthy control and psoriasis patients needs to be addressed. To date, there is no direct link between IL-17 signaling and NK cell function in psoriasis. However, NK cells have been implicated in protection from oral and dermal Candidiasis infections that requires IL-23 and IL-17 signaling (8, 141, 142). Whether NK cells participate in psoriasis via IL-17 signaling needs to be further explored. A concern about human NK cell studies is that CD56 is routinely used as a marker for NK cells, however, CD56 is also found on human IL-17 and IL-22-producing ILCs (47, 143, 144). Therefore, these studies do not exclude other CD56^+^ ILCs in the involvement in psoriasis.

The NKT frequency within the psoriatic lesions is very low— <0.1%—indicating that they are an unlikely determinant of psoriasis development (52). However, Nickoloff et al. showed that *in vitro* co-culture of NKT cells with CD1d-overexpressing keratinocytes is able to directly induce NKT production of IFNγ and IL-13. In addition, the *in vivo* injection of psoriasis lesion-derived NKT cells into the pre-psoriatic engrafted skin in SCID mice could successfully induce psoriatic plaques (54), indicating a potential role of NKT cells in the psoriasis progression. Of note, the previous attempts to use IFNγ^+^ CD3^+^/CD4^+^ T cell lines to induce psoriasis using this experimental approach were unsuccessful (145). This effect may be due to increased skin-infiltrating CD8 T cells (54), which predominantly generate IL-17 responses in human psoriasis lesions (146). This result is consistent with a human study showing that in psoriatic lesions, CD1d expression was highly enhanced in keratinocytes, which may activate the NKT cells to produce more IFNγ, thus contributing to the progression of psoriasis (55) (Figure 2). However, as previously mentioned, IL-17, TNFα, and GM-CSF production by NKT cells should also be also examined. Finally, the frequency of NKT cells expressing inhibitory receptors rather than activating receptors (CD158b^+^ and/or CD94/NKG2A^+^) was elevated in the circulation of psoriasis patients and correlated with disease severity (147). To conclude, even though they are rare in psoriatic lesions, NKT might contribute to plaque development by IFNγ production, thus recruiting more immune cells such as IL-17 producing T cells to exacerbate the disease progression.

## Conclusion

γδ T, ILC, NK, and NKT cells have all been shown to be increased in psoriasiform inflammation in humans and mice. Consistently, evidence suggests a correlation between disease severity and peripheral blood levels of γδ T, ILCs, and NKT. In addition, murine models lacking γδ T and/or ILCs demonstrated their essential role in psoriasiform inflammation development suggesting that NK and NKT cells likely play a more subtle role, a finding largely supported by studies of plaque psoriasis in humans. One fundamental characteristic of innate cells is their ability to respond rapidly and produce comparatively large amounts of inflammatory mediators in the absence of cognate antigen. Consistent with these traits, γδ T, ILCs, and NKT are all able to produce cytokines that have established pathogenicity in psoriasis. These results suggest that despite the relative rarity of these populations in psoriatic lesions, they may be more amenable to non-specific dysregulation with important consequences for disease. Interestingly, the emerging concept of “innate memory” (148), as implicated in γδ T cell-driven psoriasiform inflammation, increases the complexity of these unique leukocytes and raises new questions about their roles in complex diseases such as psoriasis.

## Author Contributions

HZ and BT wrote the manuscript. BP and IK determined the topic and wrote the manuscript.

### Conflict of Interest

The authors declare that the research was conducted in the absence of any commercial or financial relationships that could be construed as a potential conflict of interest.
